# Mechanical Strength of Thermoplastic Polyamide Welded by Nd:YAG Laser

**DOI:** 10.3390/polym11091381

**Published:** 2019-08-22

**Authors:** António B. Pereira, Fábio A. O. Fernandes, Alfredo B. de Morais, João Quintão

**Affiliations:** 1 TEMA—Centre for Mechanical Technology and Automation, Department of Mechanical Engineering, University of Aveiro, Campus de Santiago, 3810-193 Aveiro, Portugal; 2 Department of Mechanical Engineering, RISCO Research Unit, University of Aveiro, Campus Santiago, 3810-193 Aveiro, Portugal

**Keywords:** pulsed Nd:YAG laser welding, thermoplastic, polymer, polyamide 6, mechanical testing

## Abstract

Welding is a fundamental process in many industries. It is a fast-changing technology, continuously evolving, with recent developments in laser and robotic welding, virtual reality and machine learning. Focusing on laser welding, there is a significant interest in this technology, as well as an increasing demand for high-strength lightweight structures, replacing metals in some applications. This work presents an experimental study of the mechanical properties of three types of polyamide 6 joints welded by Nd:YAG laser. After welding, tensile tests were carried out in order to evaluate the influence of the joint type and weld passes on joint strength and weld stresses. The results showed that fairly high weld stresses could be achieved, indicating that good-quality welds were achieved.

## 1. Introduction

Nowadays, the combination of materials is essential in many industrial applications. Laser welding was initially applied in metals. However, with the increasing use of solid-state laser, its application was extended to polymers [1]. The number of applications of laser welding in polymers has been growing substantially, for example, in the automotive, medical, electronic and aerospace sectors. In most of these areas, high-quality, durable and reliable welds are essential, since the welds are exposed to several types of environmental conditions and subjected to different types of loads [2].

Laser welding has become a very competitive technology [1]. This relatively modern and innovative technology presents several advantages, such as no contact, high flexibility and easy handling. In particular, a solid-state laser such as the Nd:YAG laser emits a short-wavelength (1.06 μm) infrared beam, which makes it ideal for high-speed welding and robotic welding [3,4].

Thermoplastic laser welding is a relatively recent and cost-effective joining technique, with many advantages over other joining methods e.g., mechanical fastening and adhesive bonding. It allows low-weight structures and facilitates linking with other manufacturing processes. This makes it possible to reduce manufacturing times and to manufacture complex geometries [2]. It is even used in very specific applications e.g., to encapsulate high-performance polymers in medical implants [5]. The heating focused on the welded region and the absence of mechanical stresses make this technology well-suited for sensitive applications.

The joining process in laser welding is based on the melting caused by the heating associated to the radiation absorption by the base material [6]. Laser welding of thermoplastics, in particular, occurs due to the energy absorption by the material. The two parts must have different transmissive properties for the laser beam to be transmitted through one of the parts and then absorbed by the other one. The contact between both parts guarantees that the transmissive one receives heat by conduction [6,7]. In some cases, pressure is needed to guarantee full contact between samples, leading to a better heat conduction. Additionally, in order to achieve a successful weld, both polymers need to be compatible and to have similar properties, especially the melting temperature [8].

In turn, the main obstacle to laser welding thermoplastics is the high intensity of laser beams, which easily burns polymers, even at relatively low power levels. In fact, relative to metals, thermoplastics have significantly lower melting points and thermal conductivity.

There have been several experimental studies on laser welding of thermoplastics, sometimes combined with modelling to predict and optimize the process. For example, this was the approach by Van de Ven and Erdman [9] for laser welding of polyvinyl chloride (PVC) parts [9]. Later, Acherjee et al. [10] investigated the effects of process parameters, such as laser power, welding speed, laser beam diameter and clamp pressure on the lap-shear strength and the weld bead width for the laser transmission welding of PVC. New advances were also presented for the laser transmission welding of dissimilar thermoplastics [11].

Additionally, laser welding has also been used to successfully join fiber-reinforced thermoplastic composites [12,13,14,15,16]. More recently, the influence of laser welding process parameters on the weld bead quality of thermoplastic composites with high moisture content has also been studied [17]. Moisture effects on the properties of laser welded polyamide were also investigated [18].

In this work, a study of pulsed Nd:YAG laser welded joints was performed in polyamide 6 (PA 6). The joints evaluated were scarf and lap joints. The influence of the number of passes was also studied.

## 2. Materials and Methods

During previous decades, a significant number of polyamides has been developed, for instance, PA 6 and PA 66. Both present a wide range of applications, being processed and manufactured by many industries, namely the automotive sector. In fact, polyamide, a semi-crystalline thermoplastic, presents an interesting set of properties for several engineering applications [19,20] e.g., it is easy to process, has low density, is thermally stable and wear-resistant, etc. Additionally, polyamide can absorb and reflect a considerable portion of laser energy without additives, presenting good optical properties and very good welding characteristics.

In this work, the material used was PA 6, supplied by Quadrant Plastics in both white and black variants, and designated as Ertalon 6 SA. Table 1 presents the relevant properties of PA 6 for laser welding.

**Figure 1:**
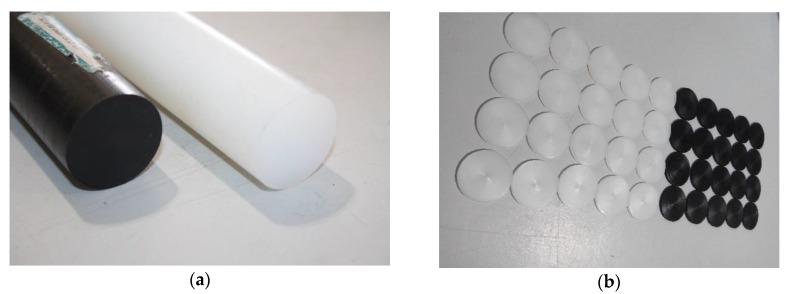
White and black Ertalon 6 SA: (**a**) rods; (**b**) discs.

The 1 m long rods supplied had 30 mm diameter (Figure 1a). The rods were first cut into 2 mm thick discs in a Pinacho lathe (Figure 1b). Finally, these were milled in a Mikron CNC machining center into 14.5 × 26 × 2 mm^3^ size parts for welding.

### 2.1. Laser Welding

The laser welding machine used was the SISMA SWA300 Nd:YAG laser, using a fundamental wavelength of 1064 nm. It has an average maximum power of 300 W and a peak pulse power of 12 kW. This machine was primarily developed for maintenance tasks such as mold repair. Nevertheless, it has been used to carry out several welding studies, from high-strength steels to dissimilar metals [21,22]. Based on the literature and after some first trials, the parameters defined in Table 2 were used to weld all the samples.

Considering the values presented in Table 2, the peak power density and the mean power density were 115 W/mm^2^ and 36 W/mm^2^, respectively. The number of pulses per diameter of focal spot is defined by a frequency of 3.84 Hz for an overlapping of 65%, as indicated in Table 2. Based on this overlapping, the cumulative dose per focal spot is 10.5 J. Regarding the focusing numerical aperture, a fixed lens aperture was defined for a distance of 105 mm between the laser output and weld bead.

#### 2.1.1. Joints

**Figure 2:**
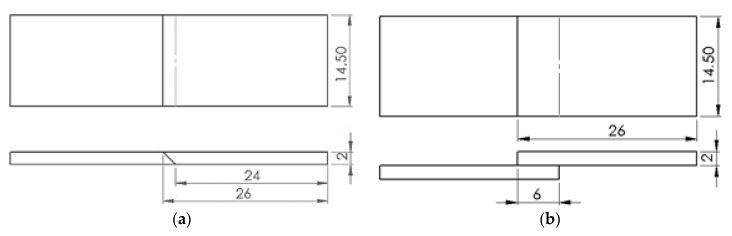
Dimensions of the types of joints welded and tested: (**a**) 45° scarf joint; (**b**) lap joint.

Figure 2 shows the dimensions of the joints welded and subsequently subjected to tensile tests. Scarf joints at 45° were performed, as well as lap joints, some of which were welded with just one pass and others with two. Three specimens of each joint type were tested.

#### 2.1.2. Clamp System

**Figure 3:**
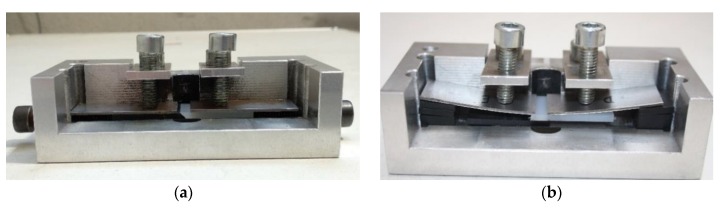
Clamp system: (**a**) butt joint sample; (**b**) lap joint sample.

In order to ensure a successful weld, both surfaces of the parts to be welded need to be kept in contact. Therefore, a clamp system was developed (Figure 3), which had two configurations adapted to the two joint types evaluated.

### 2.2. Microscopic Analysis

Before carrying out the mechanical tests, the weld beads were carefully analysed in a Mitutoyo™ optical microscope with a Moticam 2.0 photographic camera and an attached device to measure the weld dimensions.

### 2.3. Mechanical Testing

**Figure 4:**
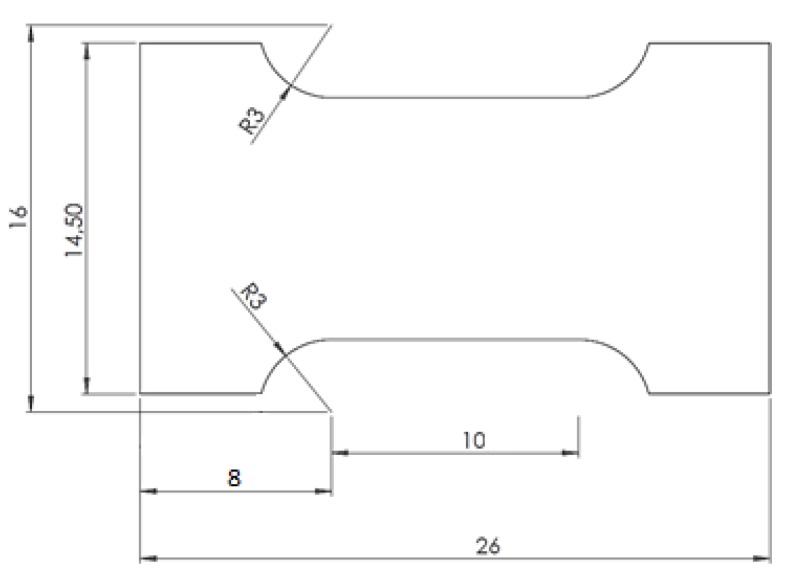
Base material sample for tensile testing.

Tensile tests were performed in order to measure the strength of the joints. The tests were carried out in a Shimadzu AGX 10 kN universal testing machine at a speed of 1 mm/min. For comparison, the base material was also tested. Figure 4 depicts the specimen dimensions, which did not follow any particular standard because of the size limitations imposed by the original rods. The results presented below show that this did not prevent realistic tensile strength values from being obtained.

## 3. Results

### 3.1. Microscopic Analysis

**Figure 5:**
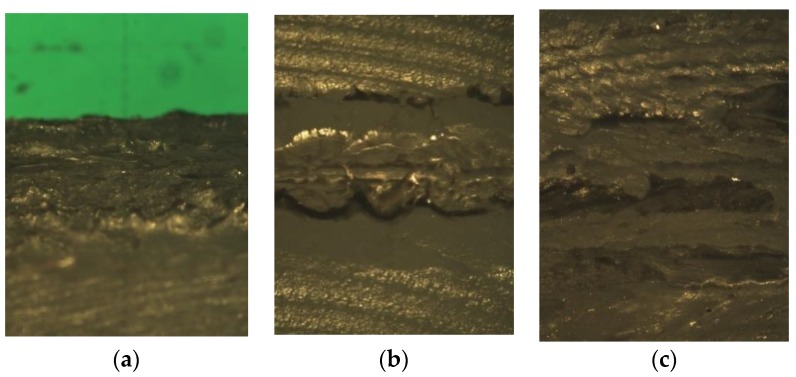
Examples of pictures of the weld beads taken during optical microscope observations: (**a**) 45° scarf joint; (**b**) single-bead lapjoint; (**c**) double-bead lap joint.

**Figure 6:**
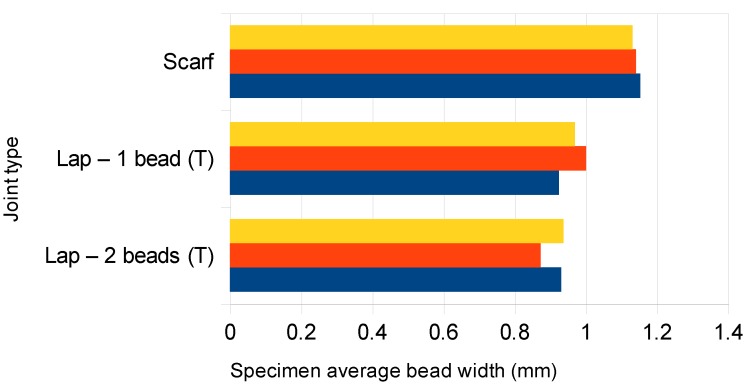
Average weld bead widths of the specimens tested.

Figure 5 presents examples of pictures of the weld beads. It was expected that 2 mm wide weld beads would be observed, considering the defined welding parameters. Nevertheless, as shown in Figure 6, the actual widths were much lower than 2 mm. This was mainly due to the reduction of the laser beam diameter after going through the transmissive part. Therefore, the constant width of the samples (14.5 mm) and the effective measured width of the weld bead of each sample were used to calculate its effective area *A_wb_*. The latter was then employed to compute an average weld failure shear stress *τ_uw_* = *F_uw_*/*A_wb_*, with *F_uw_* being the ultimate weld load. In the case of the scarf joint, simple force equilibrium gives *F_uw_* =*P_u_*cos45°, where *P_u_* is the ultimate specimen load. For lap joints, *F_uw_* =*P_u_*.

### 3.2. Mechanical Testing

#### 3.2.1. Base Material

**Figure 7:**
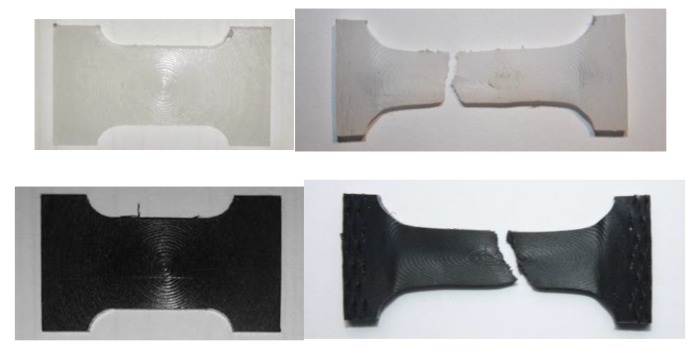
Pictures of tensile test specimens for the white and black base materials before and after the test.

All specimens failed within the gauge length and after considerable extension (Figure 7). The average tensile strengths were 67.6 MPa for the white material and 65.8 MPa for the black one. This proves that both materials are basically the same, just having different colors due to pigmentation carried out during the manufacturing process. Moreover, the present strength values agree well with those available in the literature for similar materials [23].

#### 3.2.2. Joints

**Figure 8:**
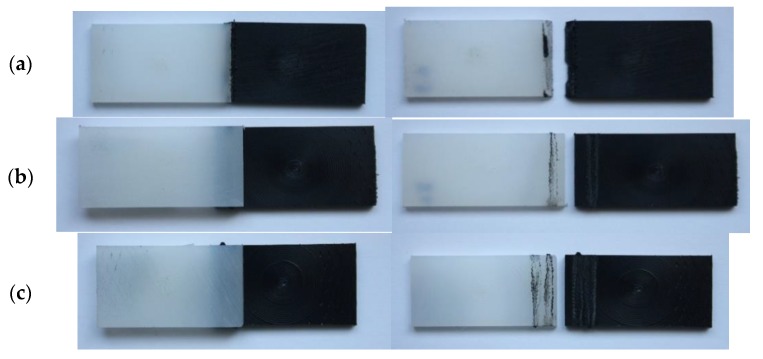
Pictures taken before and after the test of: (**a**) scarf joints; (**b**) single-bead lap joints; (**c**) double-bead lap joints.

**Figure 9:**
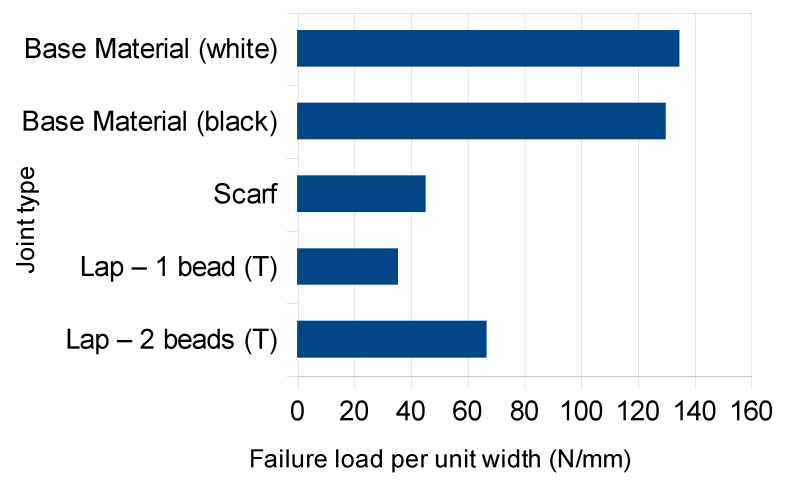
Average failure loads per unit length of the joints tested and for the base material tensile test specimens.

As exemplified in Figure 8, all joints failed at the weld beads. Figure 9 compares the failure loads per unit width. The double-bead lap joint was the strongest, reaching half of the base material failure load. Compared to the single-bead lap joint, the failure load nearly doubled with an extra bead. In turn, the scarf joint had a failure load 27% higher than that of the single-bead lap joint. This was to be expected, since scarf joints are known to be more efficient than lap joints, because of the bending caused by load eccentricity.

**Figure 10:**
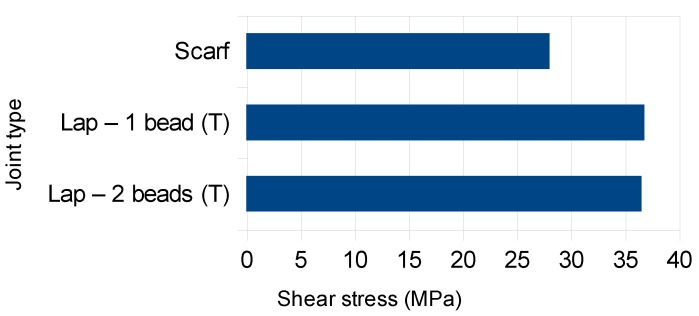
Average failure shear stress *τ_uw_* of the joints tested.

Finally, Figure 10 presents the average failure shear stress *τ_uw_* defined above. It can be seen that *τ_uw_* reached a significant 55% fraction of the base material tensile strength. This is an interesting result, considering the presence of peel stresses in lap joints and that the shear strength is lower than the tensile strength. As for the scarf joint, *τ_uw_* was naturally lower given the tensile stress acting on the weld, which is nominally identical to the shear stress.

## 4. Conclusions

In this work, scarf and lap welded joints were performed on polyamide 6. In particular, the material used was Ertalon SA 6 supplied by Quadrant Plastics in white and black versions. The welds were performed with a Nd:YAG laser. The lap joints evaluated had one or two weld passes. The base material and the joints were subjected to tensile tests. The results allow the following conclusions:

- There was a significant reduction of the laser beam diameter along the transmission through the transparent part. This resulted in considerably narrower weld beads, i.e., diameter reductions of more than 50% were observed;
- As expected, the single-weld pass scarf joint was more efficient than the single-weld pass lap joint;
- Adding one weld pass to a lap joint nearly doubled the joint strength;
- The average failure shear stress of the lap joint welds reached a considerable 55% of the base material tensile strength. This indicates good-quality welds.

To the best of our knowledge, this is the first time a Nd:YAG pulsed laser was used to perform such welds. The articles found in the literature usually refer to continuous wave lasers, such as CO_2_ lasers. Pulsed lasers make it difficult to weld, since there are cooling stages between pulses. These heating-cooling cycles, at the same spot, could cause some type of damage and defect, fortunately not observed. Therefore, this work shows that it is possible to employ a Nd:YAG pulsed laser machine, developed for metal welding, in the welding of thermoplastics. This technology makes it possible to reduce manufacturing times and to manufacture complex geometries [2], applying it to develop high performance devices from medical implants [5] to safety devices in the future (e.g., helmets [24] and vehicle structural parts [25]).

## Figures and Tables

**Table 1:** Relevant properties of Polyamide 6 for laser welding (optical properties for natural version) [6].

Density (g·m^−3^)	Refractive Index	Heat Capacity (J·K^−1^)	Thermal Conductivity (W·m^−1^·K^−1^)	Crystallinity (%)	Melting Temp. (°C)	Peak Absorbance (μm)
1.13	1.53	1.5	0.23	15–45	222	1.39

**Table 2:** Welding parameters used.

Peak Power (W)	Pulse (ms)	Overlapping (%)	Beam Diameter (mm)	Welding Speed (mm/s)
360	10	65	2	5
